# Influence of the A3669G Glucocorticoid Receptor Gene Polymorphism on the Metabolic Profile of Pediatric Patients with Congenital Adrenal Hyperplasia

**DOI:** 10.1155/2014/594710

**Published:** 2014-06-23

**Authors:** Ricardo P. P. Moreira, Larissa G. Gomes, Guiomar Madureira, Berenice B. Mendonca, Tânia A. S. S. Bachega

**Affiliations:** Unidade de Endocrinologia do Desenvolvimento, Laboratório de Hormônios e Genética Molecular (LIM/42), Disciplina de Endocrinologia, Hospital das Clínicas da Faculdade de Medicina da Universidade de São Paulo, 05403-900 Sao Paulo, SP, Brazil

## Abstract

*Background*. Pediatric CAH patients have an increased risk of cardiovascular disease, and it remains unknown if genetic predisposition is a contributing factor. Glucocorticoid receptor gene (*NR3C1*) polymorphisms are associated with an adverse metabolic profile. Our aim was to analyze the association between the *NR3C1* polymorphisms and the metabolic profile of pediatric CAH patients. *Methods*. Forty-one patients (26SW/15SV) received glucocorticoid (GC) replacement therapy to achieve normal androgen levels. Obesity was defined by BMI ≥ 95th percentile. *NR3C1* alleles were genotyped, and association analyses with phenotype were done with Chi-square, *t*-test, and multivariate and regression analysis. *Results*. Obesity was observed in 31.7% of patients and was not correlated with GC doses and treatment duration. Z-score BMI was positively correlated with blood pressure, triglycerides, LDL-c levels, and HOMA-IR. *NR3C1* polymorphisms, *Bcl*I and A3669G, were found in 23.1% and 9.7% of alleles, respectively. A3669G carriers presented higher LDL-c levels compared to wild-type subjects. *Bcl*I-carriers and noncarriers did not differ. *Conclusion*. Our results suggest that A3669G-polymorphism could be involved with a susceptibility to adverse lipid profile in pediatric CAH patients. This study provides new insight into the GR screening during CAH treatment, which could help to identify the subgroup of at-risk patients who would most benefit from preventive therapeutic action.

## 1. Introduction

Congenital adrenal hyperplasia due to 21-hydroxylase deficiency (21OHD) is a common autosomal recessive disorder caused by mutations in the* CYP21A2* gene, which encodes 21-hydroxylase (an enzyme involved in aldosterone and cortisol biosynthesis). In individuals with the disease, ACTH levels rise due to impaired cortisol secretion, thereby stimulating the adrenal cortex, accumulating androgen precursors, and resulting in varying degrees of hyperandrogenism [1–3].

The spectrum of clinical manifestations depends on the degree of enzymatic impairment. Such impairment ranges from prenatal external genitalia virilization in females and postnatal virilization in both sexes, which may occur with or without salt loss (classical forms), to a milder form with late onset hyperandrogenic signs (nonclassical) [1]. The classical forms have a prevalence of approximately one in 10,000 to one in 16,000 live births, while the nonclassical form affects approximately one in 2,500 live births [1, 2, 4].

Current CAH therapy aims to provide adequate glucocorticoid replacement and, when necessary, mineralocorticoid replacement, to avoid adrenal crisis, to suppress the increased androgen secretion (to allow the achievement of normal final height), and to avoid signs of hypercortisolism. The introduction of glucocorticoid (GC) replacement leads to significant improvement in the prognosis of classical forms. During growth periods, preference is given to the use of short half-life glucocorticoids that avoid growth suppression [1, 5, 6], and after the achievement of final height, the use of long-acting GC is suggested to allow better compliance for long-term therapy [7].

Although CAH therapy is well established [7], current limitations make it difficult to replicate the cortisol circadian rhythm, which could result in excessive glucocorticoid and/or androgen exposure.

An increased prevalence of obesity has been reported in pediatric CAH patients [8] in addition to increased lipid levels [9, 10] and insulin resistance [11]. However, it is difficult to achieve definite conclusions regarding long-term comorbidities, as previous studies included patients with classical and nonclassical forms, as well as both children and adults, all of whom require different approaches to hormonal control during CAH management.

In the general population, besides lifestyle and environmental factors, genetics variants also predispose to an adverse metabolic profile. Glucocorticoid receptor (*NR3C1*) gene polymorphisms are associated with increased cardiovascular risk, characterized by increased body mass index (BMI), blood pressure, and lipid levels [12], such as the* Bcl*I polymorphism, which is associated with increased GC sensitivity and the A3669G polymorphism linked to increased inflammatory parameters [12, 13].

In addition to the variability in the prevalence of an adverse metabolic profile among CAH patients, there are few data in the literature regarding pediatric patients. Our aim was to evaluate the influence of* NR3C1* polymorphisms on the metabolic profile in a series of pediatric CAH patients followed at the same center and undergoing similar approaches to hormone replacement.

## 2. Materials and Methods

The study was approved by the Ethical Committee of Hospital das Clínicas da Faculdade de Medicina da Universidade de São Paulo (0231/2010), and written consent was obtained from all patients' caretakers. The inclusion criteria were pediatric CAH patients with classical forms who were under stable glucocorticoid and mineralocorticoid therapy in the last two years, did not use enzyme inductor drugs, demonstrated good compliance, and had received exclusively short-acting glucocorticoids during growth periods.

### 2.1. Patients

From a cohort of 206 patients, we selected 41 children and adolescents with classical CAH, with a mean age of 11.4 ± 3.9 years (3.5 to 17.8 years). Fifteen patients (8F) had the simple virilizing form (SV), which is characterized by ambiguous genitalia at birth in girls and signs of postnatal virilization in both sexes. Twenty-six patients (16F) had the salt-wasting form (SW), and they also presented with volume depletion, sodium < 130 mmol/liter, and increased plasma renin activity (PRA) in the neonatal period. All patients presented with basal 17-hydroxyprogesterone (17-OHP) levels >150 nmol/L and molecular CAH diagnosis through entire* CYP21A2* sequence.

### 2.2. Therapy Control

Treatment efficacy was assessed by serum PRA and androgen measurements and only patients who had good compliance were selected, this being characterized by normal androgen levels according to chronological age and sex in at least 3 of 4 annual measurements and normal auxological parameters in the last two years. For prepubertal patients, testosterone and androstenedione levels were maintained ≤14 ng/dL and 2 ng/mL, respectively, and for all older patients androstenedione ≤3.5 ng/dL and testosterone levels ≤50 ng/dL for older females. Regarding mineralocorticoid replacement, the PRA levels of these patients were maintained in the upper normal limit [14]. In this period, no patient presented suppressed 17-OHP or PRA levels.

Mean daily glucocorticoid doses were calculated using body surface area (mg/m^2^) and were also evaluated retrospectively in the last 2 years. The glucocorticoid doses were converted to hydrocortisone equivalents (30 mg hydrocortisone = 37.5 cortisone acetate = 0.75 dexamethasone) [15] and presented as mg/m². Fifteen of 41 patients achieved final height (mean final height 151.4 ± 7.0; mean target height 159.7 ± 9.1), and cortisone acetate was replaced by once-daily dexamethasone (mean 0.28 ± 0.11 mg), which was available in tablet (0.5 mg) and elixir (0.1 mg/mL) formulations. For those with the salt-wasting form, fludrocortisone was maintained at a mean dose of 50 ± 25 mcg/day. The duration of therapy varied from 3.5 to 17.8 years (mean 11.4 ± 3.9 years).

### 2.3. Anthropometric and Laboratory Measurements

Children were classified as obese if their BMI was ≥95th percentile, overweight if their BMI was between the 85th and 95th percentiles, and healthy weight if their BMI was between the 5th and 85th percentiles according to age-sex tables (Centers for Disease Control and Prevention). Abnormal waist circumference was defined as circumference >90th percentile for age and sex [16]. Increased systolic or diastolic blood pressure was defined as pressures >90th percentile for age and sex [17].

Increased triglyceride levels were characterized as values ≥110 mg/dL, since all patients were less than 18 years old. Abnormal HDL-c levels were characterized as values ≤40 mg/dL, and increased impaired glucose levels were characterized as values ≥100 mg/dL. Insulin resistance was assessed by the homeostasis model assessment for insulin resistance (HOMA-IR). We also assessed HOMA-B (%; h-cell function) and HOMA-S (%; insulin sensitivity).

Metabolic syndrome was defined according to the National Cholesterol Education Program, Adult Treatment Panel III criteria (NCEP ATPIII), adapted to the pediatric group [17].

### 2.4. Genetic Analysis

PCR amplification of the glucocorticoid receptor gene regions was carried out using primer sequences and amplification conditions as previously described [18, 19]. The A3669G polymorphism was genotyped by sequencing. PCR products were sequenced using the Big Dye Terminator Sequencing KitTM (Applied Biosystem, Inc., Foster City, CA, USA) and capillary electrophoresis on an ABI PRISM 3100 sequencer (Applied Biosystem, Inc.). The* Bcl*I polymorphism was screened by an allele-specific PCR as previously described [18]. The results of the allele-specific PCR were confirmed by direct sequencing in 10 patients.

### 2.5. Statistical Analysis

Comparison of genotype frequencies between groups of patients was performed using a *x* ^2^ test. The *t*-test was applied to compare continuous variables. The results are reported as means ± SD. These analyses were performed with adjustments for age, sex, and clinical form by multivariable modeling. Pearson's correlation coefficients were used to calculate correlations between BMI z score (zBMI), GC dose, insulin, HOMA, and lipid levels after correction for age and sex. *P* < 0.05 was considered to be significant. Hardy-Weinberg equilibrium was calculated.

Statistical analysis was performed using the software SigmaStat version 3.5 for Windows (Systat Software, Point Richmond, CA).

## 3. Results

The clinical, anthropometric, and biochemical data of the 41 CAH patients are presented in Table 1. Allelic frequencies of the* Bcl*I and A3669G polymorphisms were in Hardy-Weinberg equilibrium. The* Bcl*I polymorphism was found in 23.1% of the alleles; one patient was homozygous and 18 patients were heterozygous. The A3669G polymorphism was found in 9.7% of the alleles; one patient was homozygous and 6 patients were heterozygous.

### 3.1. Clinical and Biochemical Markers of Metabolic Risk in CAH Patients

Obesity was observed in 31.7% (*n* = 13) of patients, which included 10 patients with the salt-wasting form and 6 female patients. Overweight was found in 21.9% (*n* = 9) of patients, including 4 patients with the salt-wasting form and 3 female patients. The mean GC dose in obese and normal weight patients was 14.2 ± 2.8 and 13.7 ± 4.3 mg/m²/day (*P* > 0.05), respectively, and the mean duration of therapy was 9.6 ± 5.4 and 10.9 ± 4.1 years (*P* > 0.05), respectively. There was no significant correlation with increased zBMI, sex, clinical form, or GC dose. zBMI was positively correlated with systolic blood pressure (*r* = 0.511, *P* < 0.01), HOMA-IR (*r* = 0.474, *P* < 0.01), glucose (*r* = 0.322, *P* < 0.01), total cholesterol (*r* = 0.429, *P* < 0.01), triglycerides (*r* = 0.422, *P* < 0.01), and LDL-c levels (*r* = 0.430, *P* < 0.01).

Interestingly, SW patients presented a worse lipid profile in comparison with SV patients, as characterized by increased total cholesterol, LDL-c, and TG levels, which were independent of zBMI. Blood pressure and HOMA values did not differ between patients with the SW and SV forms (Table 1).

At the time of this analysis, 15 patients achieved final height and were receiving dexamethasone. The regression analysis did not show differences in the clinical or metabolic profile between patients receiving cortisone acetate and dexamethasone.

### 3.2. Influence of NR3C1 Polymorphisms on the Metabolic Profile of Pediatric CAH Patients

A comparison of the metabolic profile between carriers and noncarriers of the A3669G polymorphism is shown in Table 2. A3669G carriers had higher LDL-c levels compared to wild-type carriers in the *t*-test analysis, which maintained significance after adjustment by sex, age, and clinical form (Table 2). There were no significant differences observed in the HOMA value and blood pressure between carriers and noncarriers of the A3669G polymorphism. There was no significant difference in the frequency of the A3669G polymorphism between the obese and nonobese CAH patients, 42.8%* versus* 29.4%, respectively, between the patients with and without metabolic syndrome, 14.3%* versus* 14.7%, respectively, or between the patients with and without hypertension, 14.3%* versus* 8.8%, respectively.

Table 3 shows the metabolic profile of the* Bcl*I and wild-type carriers. Although* Bcl*I carriers showed a tendency to adverse metabolic profile, these differences were not statistically significant (Table 3). There was no significant difference in the frequency of the* Bcl*I polymorphism between the obese and nonobese CAH patients, 33.3%* versus* 30.4%, respectively, between the patients with and without metabolic syndrome, 22.2%* versus* 8.7%, respectively, or between the patients with and without hypertension, 16.7%* versus* 4.3%, respectively.

## 4. Discussion

The increased prevalences of obesity and adverse metabolic profiles are important factors in the development of cardiovascular disease. Some studies have described abnormal body compositions and unfavorable trends in metabolic risk in CAH adults [9, 20–23]; however, data regarding the metabolic profile of the pediatric CAH are limited.

Similar to what was observed in the general population, in our series of pediatric CAH patients, the zBMI and waist-hip ratio were positively correlated with blood pressure, an adverse lipid profile, and insulin resistance. We did not observe differences in the metabolic profile of CAH patients receiving dexamethasone compared to those receiving cortisone acetate.

Surprisingly, we observed that SW patients presented worse clinical and metabolic profiles compared to SV patients, independent of age and sex. These results could be related to the earlier treatment onset in SW patients. In addition, a tendency to overtreat SW patients in the first year of life cannot be ruled out.

The main cause of increased obesity in CAH patients is related to chronic glucocorticoid exposure. However, as occurs in the general population, environmental factors and genetic predisposition may contribute to increased obesity frequency in this population [24, 25], and these factors have not been evaluated among patients with CAH. Recently, we described the influence of the* NR3C1*-*Bcl*I polymorphism on the prevalence of obesity in adult CAH patients [26]. However, in this pediatric CAH cohort, despite zBMI and waist circumferences being higher in* Bcl*I carriers than wild type carriers, these differences were not statistically significant and we cannot rule out a sample size effect. Therefore, we tested the influence of* NR3C1* polymorphisms on the metabolic profiles of pediatric CAH patients and found a positive association between the presence of the A3669G allele and higher LDL-c levels.

Our results are in line with previous data from the general population, as our patients carrying the A3669G allele presented with a poor metabolic profile, independent of sex, age, and clinical form (Table 2). This polymorphism is associated with increased expression and stabilization of the 9*β* GR-isoform and a recent study suggested that it is associated with increased cardiovascular risk in comparison to noncarriers, which was supported by the findings of elevated levels of inflammatory parameters in a large cohort of elderly subjects [13]. However, the main limitation of this study is the sample size, which was restricted by our attempt to select a homogenous CAH cohort.

In conclusion, our findings suggest that the A3669G polymorphism is involved with a susceptibility to an adverse lipid profile in pediatric CAH patients. Therefore, this study provides new insight into the GR screening during CAH treatment, which could help to identify the subgroup of at-risk patients who would most benefit from preventive therapeutic action. However, despite our cohort having a good sample size considering the disease frequency, these data should be reevaluated in future multicentric studies, focusing on these polymorphisms and their possible influence on clinical phenotypes of CAH patients under GC replacement.

## Figures and Tables

**Table 1:** Clinical and anthropometric characteristics of 41 pediatric CAH patients.

Variables	Salt wasters (*n* = 26)	Simple virilizing (*n* = 15)	*P* value
Age, mean (SD), years	10.6 (4.1)	12.8 (3.4)	0.090
Obesity, *n* (%)	10 (38.4)	3 (20)	0.381
Metabolic Syndrome, *n* (%)	4 (15.4)	2 (13.3)	0.780
GC dose, mean (SD), mg/m^2^	9.6 (2.9)	11.1 (4.9)	0.428
Duration of GC therapy, mean (SD), years	10.7 (4.5)	9.3 (4.0)	0.423
*z*BMI, mean (SD), kg/m^2^	1.04 (1.09)	0.90 (0.74)	0.658
Waist circumference, mean (SD), cm	66.4 (11.0)	70.8 (7.3)	0.177
Hip, mean (SD), cm	80.3 (12.9)	88 (9.1)	0.052
Hip-waist ratio, mean (SD)	0.83 (0.06)	0.80 (0.04)	0.207
Increased waist circumference, (SD), *n* (%)	13 (50)	8 (53.3)	0.906
Fasting glucose, mean (SD), mg/dL	82.4 (8.1)	85.1 (10.7)	0.368
Fasting insulin mean (SD), *μ*lU/mL	12.7 (9.5)	13.9 (10.8)	0.548
HOMA-IR, mean (SD)	1.58 (1.15)	1.73 (1.30)	0.547
HOMA-S, mean (SD)	94.7 (58.7)	82.1 (50.1)	0.494
HOMA-B, mean (SD)	155.0 (75.8)	157.9 (78.8)	0.845
Total cholesterol, mean (SD), mg/dL	159.2 (33.7)	136.6 (23.7)	*0.033 *
HDL-c, mean (SD), mg/dL	44.2 (12.8)	51.0 (56.6)	0.163
LDL-c, mean (SD), mg/dL	99.8 (30.7)	75.2 (12.3)	*0.006 *
Triglycerides, mean (SD), mg/dL	76.4 (33.0)	52.4 (17.0)	*0.014 *
Increased blood pressure, *n* (%)	2 (7.7)	2 (13.3)	0.968

**Table 2:** Influence of A3669G polymorphism on the metabolic profile of CAH patients.

Variables	Genotypes		*P* value^a^	Adjusted *P* value
	A3669G *n* = 7	Wild type *n* = 34		
Obesity, *n* (%)	3 (42.8)	10 (29.4)	0.802	0.568
Metabolic syndrome, *n* (%)	1 (14.3)	5 (14.7)	0.576	0.819
GC dose, mean (SD), mg/m²	7.7 (3.5)	10.5 (3.9)	0.085	0.125
Duration of GC therapy, years	11.1 (4.2)	9.7 (5.0)	0.542	0.657
*z*BMI, mean (SD)	1.63 (0.66)	0.77 (1.0)	0.037	0.226
Waist circumference, mean (SD), cm	72.0 (6.6)	67.8 (10.6)	0.331	0.089
Hip, mean (SD), cm	85.9 (10.8)	82.7 (12.4)	0.540	0.133
Hip-waist ratio, mean (SD)	0.84 (0.08)	0.81 (0.05)	0.176	0.279
Increased waist circumference, (SD), *n* (%)	6 (85.7)	15 (34.1)	0.112	0.208
Fasting glucose, mean (SD), mg/dL	79.7 (3.4)	84.2 (9.8)	0.193	0.327
Fasting insulin mean (SD), *μ*lU/mL	12.2 (5.9)	13.4 (10.6)	0.656	0.804
HOMA-IR, mean (SD)	1.51 (0.71)	1.67 (1.28)	0.695	0.787
HOMA-S, mean (SD)	76.1 (27.2)	92.9 (59.5)	0.469	0.578
HOMA-B, mean (SD)	162.8 (49.6)	154.7 (80.9)	0.444	0.797
Total cholesterol, mean (SD), mg/dL	162.6 (45.2)	147.2 (29.8)	0.281	0.237
HDL-c, mean (SD), mg/dL	39.1 (7.5)	48.3 (15.2)	0.132	0.176
LDL-c, mean (SD), mg/dL	110.9 (38.3)	85.3 (23.2)	0.029	*0.021 *
Triglycerides, mean (SD), mg/dL	62.9 (23.3)	69.7 (34.2)	0.726	0.633
Increased blood pressure, *n* (%)	1 (14.3)	3 (8.8)	0.798	0.752

Values are given as means ± SD.

Adjusted for age, sex, and clinical form.

^a^Univariated analysis.

**Table 3:** Influence of *Bcl*I polymorphism on the metabolic profile of CAH patients.

Variables	Genotypes		*P* value^a^	Adjusted *P* value
	*Bcl*I *n* = 20	Wild type *n* = 21		
Obesity, *n* (%)	6 (33.3)	7 (30.4)	0.888	0.120
Metabolic syndrome, *n* (%)	4 (22.2)	2 (8.7)	0.441	0.359
GC dose, mean (SD), mg/m^2^	10.3 (4.0)	9.4 (4.4)	0.561	0.614
Duration of GC therapy, mean (SD), years	10.9 (4.8)	9.4 (4.6)	0.464	0.880
*z*BMI, mean (SD)	1.01 (1.06)	0.8 (0.91)	0.549	0.541
Waist circumference, mean (SD), cm	69.6 (11.5)	67.9 (6.5)	0.630	0.553
Hip, mean (SD), cm	84.6 (13.4)	84.1 (9.1)	0.903	0.825
Hip-waist ratio, mean (SD)	0.82 (0.07)	0.81 (0.04)	0.484	0.421
Increased waist circumference, (SD), *n* (%)	9 (50)	12 (52.1)	0.860	0.725
Fasting glucose, mean (SD), mg/dL	84.7 (11.1)	82.7 (8.3)	0.890	0.570
Fasting insulin mean (SD), *μ*lU/mL	11.4 (6.6)	15.3 (10.6)	0.221	0.263
HOMA-IR, mean (SD)	1.43 (0.82)	1.91 (1.27)	0.219	0.248
HOMA-S, mean (SD)	91.7 (49.4)	72.8 (39.8)	0.233	0.230
HOMA-B, mean (SD)	141.8 (61.7)	175.2 (75.4)	0.165	0.222
Total cholesterol, mean (SD), mg/dL	153.7 (42.4)	146.9 (22.8)	1.00	0.827
HDL-c, mean (SD), mg/dL	46.3 (12.0)	45.2 (15.8)	0.601	0.836
LDL-c, mean (SD), mg/dL	93.2 (37.3)	89.2 (16.7)	0.732	0.700
Triglycerides, mean (SD), mg/dL	70.9 (36.1)	63.5 (27.5)	0.652	0.938
Increased blood pressure, *n* (%)	3 (16.7)	1 (4.3)	0.430	0.324

Values are given as means ± SD.

Adjusted for age, sex, and clinical form.

^a^Univariated analysis.'
